# Effects of Semisupervised Exercise Training on Health Outcomes in People With Lung or Head and Neck Cancer: Protocol for a Randomized Controlled Trial

**DOI:** 10.2196/43547

**Published:** 2023-05-24

**Authors:** Isis Grigoletto, Vinicius Cavalheri, Luis Alberto Gobbo, Karina Pozo, Enio Rodrigues Maia Filho, Diogo Gonçalves Ribeiro, Nara Ielo, Fabiano Francisco De Lima, Ercy Mara Cipulo Ramos

**Affiliations:** 1 Department of Physiotherapy, Faculty of Science and Technology São Paulo State University Presidente Prudente, São Paulo Brazil; 2 Cancer Hospital of Presidente Prudente Presidente Prudente, São Paulo Brazil; 3 Curtin School of Allied Health, Faculty of Health Sciences Curtin University Perth, Western Australia Australia; 4 Curtin enAble Institute, Faculty of Health Sciences, Curtin University Perth, Western Australia Australia; 5 Allied Health, South Metropolitan Health Service Perth, Western Australia Australia; 6 Department of Physical Education, Faculty of Science and Technology São Paulo State University Presidente Prudente, São Paulo Brazil; 7 São Leopoldo Mandic Institute and Research Center Campinas, São Paulo Brazil; 8 Department of Physiotherapy, Speech and Occupational Therapy School of Medicine, University of São Paulo São Paulo Brazil

**Keywords:** lung neoplasms, head and neck neoplasms, drug therapy, radiotherapy, exercise, muscle strength, lung cancer, neck cancer, head cancer, aerobic exercise, pulmonary, neoplasm, ENDT, ear nose throat, ear, nose, and throat, RCT, oncology, outpatient, cancer treatment, Eastern Cooperative Oncology Group, ECOG, HRQoL, quality of life, QoL, physical activity

## Abstract

**Background:**

Lung or head and neck cancers are known for their high prevalence and mortality rates. Chemotherapy and radiotherapy are usually recommended as cancer treatment for these malignancies; however, they can negatively impact both the physical and mental status of patients. Hence, it is reasonable to consider resistance and aerobic exercise training to prevent these negative health outcomes. Further, several factors prevent patients from attending outpatient exercise training programs, and, therefore, a semisupervised home-based exercise training program may be seen as a well-accepted alternative.

**Objective:**

The aim of this study will be to investigate the effects of a semisupervised home-based exercise training program on physical performance, body composition, and self-reported outcomes; changes in the initial cancer treatment dose prescribed; number of hospitalizations at 3, 6, and 9 months; and 12-month survival in people with primary lung or head and neck cancer.

**Methods:**

Participants will be randomly allocated to the training group (TG) or control group (CG). The TG will undergo semisupervised home-based resistance and aerobic exercise training throughout their cancer treatment. The resistance training will be performed using elastic bands (TheraBand) twice a week. The aerobic training (ie, brisk walk) will be performed for at least 20 minutes per day outdoors. The equipment and tools used during the training sessions will be provided. This intervention will start the week before treatment commencement, will be performed throughout the duration of the treatment, and will continue for 2 weeks after treatment completion. The CG will undergo usual care (ie, cancer treatment with no formal exercise prescription). Assessments will take place 2 weeks before the beginning of the usual cancer treatment and 2 weeks after treatment completion. The measures of physical function (peripheral muscle strength, functional exercise capacity, and physical activity), body composition, and self-reported outcomes (symptoms of anxiety and depression, health-related quality of life, and symptoms related to the disease and treatment) will be collected. We will report on any change in the initial cancer treatment dose prescribed; number of hospitalizations at 3, 6, and 9 months; and 12-month survival.

**Results:**

In February 2021, the clinical trial registration was approved. Recruitment and data collection for the trial are ongoing (as of April 2023, 20 participants had already been randomized), and findings of this study are likely to be published late in 2024.

**Conclusions:**

This exercise training as a complementary treatment for patients with cancer is likely to promote positive effects on the health outcomes assessed, over and above any change in the CG, and prevent the reduction of initial cancer treatment dose prescribed. If these positive effects are shown, they will likely impact long-term outcomes such as hospitalizations and 12-month survival.

**Trial Registration:**

Brazilian Clinical Trials Registry (ReBEC) RBR-5cyvzh9; https://ensaiosclinicos.gov.br/rg/RBR-5cyvzh9.

**International Registered Report Identifier (IRRID):**

PRR1-10.2196/43547

## Introduction

### Background

Worldwide, lung or head and neck cancers are the most common cancers and the main causes of death each year [1,2]. Because of the direct effect of these cancers on the respiratory system, management and rehabilitation may be substantially similar [3]. The usual cancer treatment options are surgery, chemotherapy, radiotherapy, or a combination of these treatments [4]. Although having positive effects, these treatments also have side effects, such as fatigue [5,6] and a decrease in functional exercise capacity [7], muscle mass [8], and muscle strength [8,9], which will ultimately affect physical activity and health-related quality of life (HRQoL) [10,11]. Therefore, complementary interventions that could minimize these side effects are needed [7].

Studies have demonstrated that exercise promotes benefits to this population [7]. Improvements have been reported in physical function, body composition, psychosocial function, sleep quality, fatigue, HRQoL [12], and muscle strength [7] after completion of an exercise program. When used as a complementary treatment to usual cancer treatment, exercise has been shown to maximize chemotherapy completion in women with breast cancer and men with prostate cancer [13,14].

Exercise training has been shown to be safe and have positive effects on health outcomes in people with lung or head and neck cancer [15]. Taking into account these positive effects [16,17], as well as the fact that exercise is a low-cost treatment modality that attenuates symptoms related to cancer treatment, it is imperative that exercise training is delivered as a complement or adjunct to medical treatment [15-17].

Distance- and travel-related costs are factors that influence the patient’s enrollment and attendance to exercise, and home-based exercises seem to be an option that can overcome these barriers. Although being a feasible alternative [18-20], home-based exercises should be semisupervised to ensure safety and adherence, as well as maximize training efficacy. As there is limited data on the effects of a semisupervised home-based exercise program in people with lung or head and neck cancer, this randomized controlled trial aims to investigate the protective effect of semisupervised home-based exercise training on physical function, body composition, and self-reported outcomes in people with primary lung or head and neck cancer. In addition, we will also report on changes in the initial cancer treatment dose prescribed; number of hospitalizations at 3, 6, and 9 months; and 12-month survival.

### Hypotheses

We hypothesize that the semisupervised home-based exercise training as a complementary treatment will promote positive effects on physical function, body composition, and self-reported outcomes in these patients during the cancer treatment. It is also expected that the initial usual cancer treatment dose prescribed will be maintained because of the benefits of the exercise training, leading to fewer hospitalizations and greater survival in patients who underwent the exercise training program.

## Methods

### Study Design

This will be a single-blind (outcome assessor), 2-arm randomized controlled trial. The study will be conducted at a cancer hospital. The study protocol has been developed following the SPIRIT (Standard Protocol Items: Recommendations for Interventional Trials) 2013 checklist guidelines.

### Ethics Approval

The trial has received approval from the health research ethics committee of the Regional Cancer Hospital of Presidente Prudente (HRCPP; 26123119.5.0000.8247), which complies with the Declaration of Helsinki. Written, informed consent to participate will be obtained from all participants.

### Data Collection and Management

The information collected about participants is individually identifiable by members of the research team only. Each participant will be allocated a unique numeric code (ID number) such that all stored electronic data (eg, databases and data files) will not contain identifiable data until the completion of the study. All confidential information is stored in locked filing cabinets, and only deidentified data will be presented or published.

### Study Population

Participants will be recruited from the outpatient clinic before cancer treatment commencement. The inclusion criteria will be as follows: diagnosis of primary lung or head and neck cancer; older than 18 years; referred for cancer treatment including radiotherapy, chemotherapy, or both; Eastern Cooperative Oncology Group performance status score between 0 and 2; life expectancy (physician-rated) of >6 months; and sufficient functional literacy or having a support person who can help with reading the instructions for the home-based exercise program. The exclusion criteria will be as follows: people referred for cancer surgery or palliative care; symptomatic brain or bone metastasis prohibiting participation in exercise training; and unstable metabolic, musculoskeletal, or cardiovascular disease.

### Experimental Design

**Figure 1:**
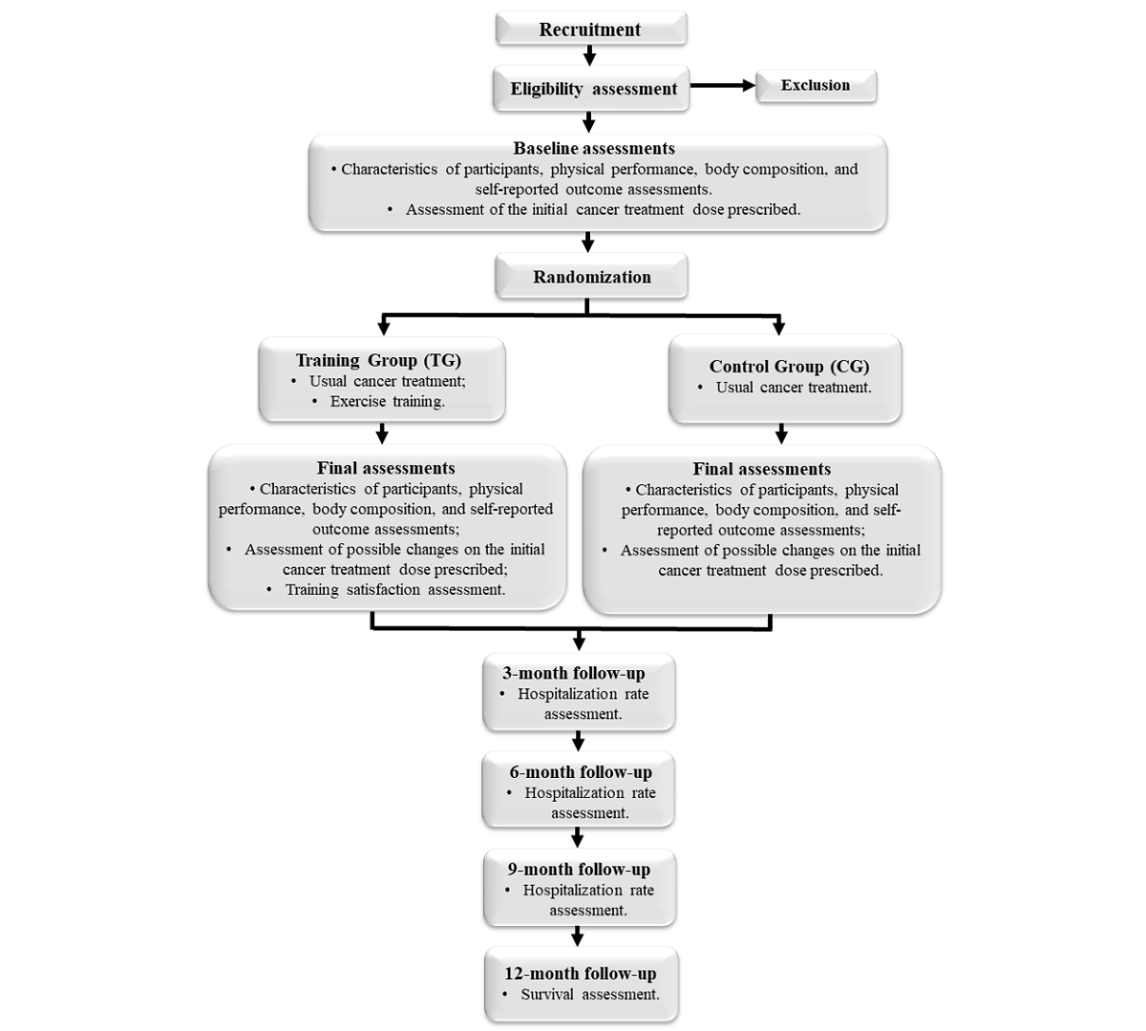
Experimental design of the study.

The flow of participants through the study is presented in Figure 1, and the overview of the data collection is given in Table 1. First, people referred to usual lung or head and neck cancer treatment (chemotherapy or radiotherapy) will be assessed on 2 nonconsecutive days. On assessment day 1, participants’ characteristics and past medical history will be collected, and data on physical activity, symptoms of anxiety and depression, HRQoL, and symptoms related to treatment and disease will be assessed via validated questionnaires. Subsequently, peripheral muscle strength will be assessed via digital dynamometry. On assessment day 2, measures of body composition (octapolar Inbody 720; Biospace) and functional exercise capacity will be undertaken, including the 6-minute walk test (6MWT), time up and go test (TUG), and 1-minute sit-to-stand test (1STS). After assessment day 2, participants will be randomly allocated to either the training group (TG) or the control group (CG). A third visit will be scheduled for those allocated to the TG, where the exercise training protocol will be explained and demonstrated, and the participants will be familiarized with the training equipment and tools. Participants in the CG group will undergo usual cancer treatment (chemotherapy and radiotherapy) and will be instructed to keep their routine activities during the study period. They will also receive phone calls every 15 days (throughout the course of their cancer treatment) to discuss any issues related to cancer treatment and their participation in the study.

**Table 1:** Overview of the information collected at baseline, during the intervention, at the end of the study, and at the follow-ups.

Outcome	Assessment	Baseline	During intervention	Final	Follow-up (3, 6, and 9 months)	Follow-up (12 months)
Characteristics of the participants	By interview	✓		✓		
Functional capacity	6MWT^a^	✓		✓		
Functional capacity	TUG^b^	✓		✓		
Functional capacity	1STS^c^	✓		✓		
Physical activity	IPAQ^d^	✓		✓		
Body composition	Bioimpedance spectroscopy (octopolar InBody 720; Biospace)	✓		✓		
Anxiety and depression	HADS^e^	✓		✓		
HRQoL^f^	EORTC QLQ-30^g^/QLQ-LC13	✓		✓		
Symptoms related to the treatment and disease	MSAS-BR^h^					
Cancer treatment dose prescribed	Via the participant’s medical records	✓				
Cancer treatment dose completed	Via the participant’s medical records			✓		
Hospitalization rate assessment	Via telephone calls				✓	
Survival rate assessment	Via telephone calls					✓

^a^6MWT: 6-minute walk test.

^b^TUG: time up and go test.

^c^1STS: 1-minute sit-to-stand test.

^d^IPAQ: International Physical Activity Questionnaire.

^e^HADS: Hospital for Anxiety and Depression Scale.

^f^HRQoL: health-related quality of life.

^g^EORTC QLQ-30: European Organization for Research and Treatment of Cancer Core Quality of Life Questionnaire.

^h^MSAS-BR: Memorial Symptom Assessment Scale (Portuguese version).

Two weeks after completion of the cancer treatment, participants will be reassessed (ie, repetition of the 2 days of assessments described above). Data on changes in the initial cancer treatment dose prescribed will be collected during the intervention period. Participants (or their support person) will receive phone calls at 3, 6, 9, and 12 months after the reassessment date. The number of hospitalizations at 3, 6, and 9 months as well as 12-month survival will also be recorded.

### Randomization, Concealment, and Blinding

The randomization sequence will be generated on a web-based platform [21] and concealed using opaque envelopes. This process will be conducted by a researcher who is not involved with participant recruitment, assessment, or data management and analysis. The assessor will be blinded to group allocation. Assessments and patient recruitment will be blinded to the therapeutics.

### Procedures (TG and CG Intervention)

Participants in both groups (TG and CG) will undergo usual cancer treatment that does not include any formal exercise prescription. Those allocated to the TG will perform resistance and aerobic exercises that will be individually prescribed.

#### TG Intervention

The semisupervised home-based exercise training program will start 1 week before commencement of cancer treatment and will finish 2 weeks after completion of the cancer treatment. Semisupervision will include an exercise diary, daily SMS text messaging, and frequent phone calls. A previous study [22] reported that people who underwent semisupervised home-based exercise training did not have any adverse event during the training program. The resistance and aerobic training will be guided by an exercise diary developed by the research team; see Multimedia Appendix 1 for more details. Participants in the TG will be instructed to complete the diary on a daily basis with information on the exercises they performed. They will also be provided with a pulse oximeter (G-Tech Portable Oled) and the modified Borg scale to assess and record their peripheral capillary oxygen saturation and the Borg dyspnea score, respectively, before and after the exercise training session. Participants will be asked to avoid exercise training if resting oxygen saturation is <90%. Resistance training will be performed twice a week and aerobic training will be performed 7 days a week. Participants will also receive a daily SMS text message reminding them to perform the exercises and will be contacted (via a phone call) every 7 to 14 days.

To ensure participants’ safety during the exercise program, blood tests will be performed every second week to monitor platelet count and hemoglobin levels. Exercise training will be paused when there is fever (>38 °C) [23], infection, hemoglobin level <8 g/dL [24,25], and psychological instability or clinical complications [26].

##### Resistance Training

Participants in the TG will perform semisupervised home-based resistance training using elastic bands (TheraBand, green color, moderate resistance) [26]. Exercises for the following muscle groups will be included: elbow flexors, knee flexors, and knee extensors (2 sets of 10 repetitions with a 1-minute interval) [27]. The exercise intensity will be adjusted using the modified Borg scale (the participant must maintain dyspnea between 4 and 6, ie, moderate intensity) [22]. Regarding progression of the exercise prescription, if the participant is able to perform more than 2 sets of 10 repetitions and report a Borg dyspnea score <4, an increment of 5 repetitions will be prescribed for the following session using the same elastic band resistance [28]. Subsequently, the intensity progression will be to 3 sets of 10 repetitions and then 3 sets of 15 repetitions. Participants will be contacted via a phone call (or in person, if needed) every 7 to 14 days to assess the need for increased training intensity. This protocol was developed based on previous studies of exercise training in people with cancer [22,26-28] as well as of resistance training with elastic bands in people with chronic obstructive pulmonary disease [29,30].

##### Aerobic Training

For the aerobic training, participants will perform a brisk walk outside, starting with 20 minutes daily (equivalent to 1.5 km) [28], and will be asked to maintain a Borg dyspnea score between 4 and 6 (moderate intensity) [31]. Therefore, this training will be equivalent to approximately 3.5 metabolic equivalents [28]. Participants will be provided with a pedometer (Power Walk PW-610; Yamax) as a feedback tool for the aerobic training. Participants will be instructed to try to achieve at least 150 minutes of aerobic training weekly. The modified Borg scale will also be used to titrate the intensity of the aerobic training (if symptoms of dyspnea are <4 on the Borg scale, an extra 5 minutes will be prescribed for the following week). If more than 150 minutes of weekly aerobic training is achieved, the participants will be instructed to increase their walking speed but maintain a Borg dyspnea score between 4 and 6. This protocol has been developed based on previous studies of physical training for people with cancer [26,28].

#### CG Intervention

Participants in the CG will undergo usual cancer treatment with no formal exercise prescription and will be instructed to keep routine activities during the study period. They will also receive phone calls every 15 days to discuss any issues related to cancer treatment and their participation in the study.

### Outcome Measures (Assessments)

#### Characteristics of Participants

Participant characteristics will be collected of age, gender, height, body weight, cancer type and stage, number of cycles per session as well as dose of chemotherapy or radiotherapy prescribed, and self-reported comorbidities.

#### Primary Outcome

##### Peripheral Muscle Strength

Peripheral muscle strength will be assessed via the force gauge digital dynamometer (Instrutherm DD-300), and the results will be expressed in newtons. The participants will be instructed to perform elbow flexion (at 180°), knee flexion (at 90°), and extension (at 90°) movements resisted by a steel cable coupled to the dynamometer. The measurement will be repeated 5 times with an interval of 1 minute between attempts, and the highest value among the 3 closest measurements will be recorded [32].

#### Secondary Outcomes

##### Functional Exercise Capacity

Functional exercise capacity will be assessed via the 6MWT and the 1STS. The 6MWT will be performed in accordance with the guidelines established by the American Thoracic Society and European Respiratory Society [33]. Two tests will be performed, with at least 30 minutes of rest between them, and the best performance (ie, 6-minute walk distance) will be reported. The reference values developed for the Brazilian population will be used [34].

The 1STS test will also be used to evaluate the functional exercise capacity. Two tests will be performed, with 1 minute of rest between them, and best performance (ie, number of sit-to-stand repetitions) will be reported [35].

##### Mobility

Mobility and the capacity to perform routine activities will be assessed by the TUG. The TUG has also been used to assess risk of falls, and the performance in the TUG is determined by the time spent to complete the test [36,37].

##### Physical Activity

The International Physical Activity Questionnaire will be used to assess self-reported physical activity. The variable from the International Physical Activity Questionnaire that will be used is time spent in physical activity [38].

##### Body Composition

Body composition will be assessed via bioimpedance spectroscopy (octopolar InBody 720; Biospace) following the manufacturer’s instructions. Data will be electronically imported to Excel (Microsoft Corp) using the software Lookin’Body (version 3.0; Biospace) [39]. The following variables will be assessed: intracellular water, extracellular water, total body water, basal metabolic rate, protein mass, skeletal muscle mass, bone mineral content, fat mass, and body weight.

##### Symptoms of Anxiety and Depression

Symptoms of anxiety and depression will be assessed via the Hospital for Anxiety and Depression Scale. This scale comprises 14 questions, divided into 2 subscales: anxiety and depression. The final score indicates probably not anxiety or depression, questionable or doubtful anxiety or depression, and probable anxiety or depression [40].

##### Health-Related Quality of Life

HRQoL will be assessed using the European Organization for Research and Treatment of Cancer Core Quality of Life Questionnaire (EORTC QLQ-30) [41]. The specific module for lung cancer (QLQ-LC13) [42] will also be used. Higher scores in physical scales and global health scales indicate greater HRQoL, whereas higher scores in symptoms scales indicate worse symptoms and poorer HRQoL [41].

##### Symptoms Related to the Treatment and Disease

The Memorial Symptom Assessment Scale (Portuguese version) will be used to assess symptoms related to the treatment and disease. This scale comprises items related to psychological and physical symptoms [43].

### Other Measures

Any change in the initial cancer treatment dose prescribed will be investigated via the participant’s medical records. Participants (or their support person) will receive phone calls at 3, 6, 9, and 12 months after the reassessment date. The number of hospitalizations at 3, 6, and 9 months as well as 12-month survival will also be recorded. Satisfaction with the semisupervised home-based exercise training program will be assessed using a questionnaire that comprises 2 questions [44]. Participants will rate, on a scale ranging between 0 and 10, their satisfaction with performing the exercise training program and how valuable the exercise training program was for them. Higher scores indicate greater satisfaction.

### Sample Size Calculation

The sample size was determined using published data on the effectiveness of exercise training in improving peripheral muscle strength in patients with cancer [45]. Forty participants will be needed to detect a between-group difference of 72 (SD 71) newtons on the specific primary outcome: knee extensors’ muscle strength [46] (power of 80%, adopting an α of 5%). This calculation accounts for 30% loss to follow-up.

### Data Management and Analysis

The statistical software SPSS (version 23.0; IBM Corp) will be used. The distribution of the data will be assessed by the Shapiro-Wilk test. Continuous data will be expressed as either mean (SD) or median (IQR) according to their distribution. The criteria that define the exclusion of the participant from the final data analysis will be (1) initiation of palliative care or (2) substantial physical impairment due to hospitalization during the intervention period. For data that are normally distributed, both within- and between-group differences will be assessed using the 2-way repeated measures ANOVA. Between-group differences will be reported as the mean difference and 95% CI [47,48]. For data that are not normally distributed, within-group differences will be assessed using the Wilcoxon test, whereas between-group differences will be assessed using the Mann-Whitney *U* test. To evaluate the effect size between groups, the Cohen *d* test will be used. For all analyses, a *P* value of <.05 will be considered significant. The within-group change from baseline to postintervention will be assessed via either a 2-tailed paired *t* test or the Mann-Whitney *U* test. The chi-square test will be used to analyze hospitalization frequency at 3, 6, and 9 months, and the hazard ratio will be used to report on 12-month survival. Analyses will be undertaken according to the intention-to-treat principle.

## Results

In February 2021, the clinical trial registration was approved. Recruitment and data collection for the trial are ongoing, and the results of this study are likely to be published late in 2024.

## Discussion

### Potential Impact and Significance of the Study

This study protocol has been developed after a thorough literature search on exercise training for people with cancer, specifically lung or head and neck cancers. The implementation of this novel exercise training protocol, which will be based on a semisupervised home-based program delivered as a complementary treatment in people with lung or head and neck cancer, will investigate whether the program will promote a protective effect on physical function, body composition, and self-reported outcomes. Further, the study will also explore the effects of the program on any change in initial cancer treatment dose prescribed; hospitalizations at 3, 6, and 9 months; and 12-month survival.

### Strengths and Limitations of the Study

This study will be a single-blinded (outcome assessor) randomized controlled trial in which participants allocated to the TG will undergo an individualized home-based exercise program. The study will be the first to include both people with lung cancer and those with head and neck cancer. In addition to reporting the effectiveness of the proposed intervention on important health outcomes, this novel study will also explore the effects of the proposed intervention on any change in the initial cancer treatment dose prescribed; hospitalizations at 3, 6, and 9 months; and the 12-month survival rate. As in any study in the field of exercise training, blinding of participants and personnel is very challenging, and in this case, it is not possible. The study will only be conducted in a single center, which may limit its external validity. However, the robust reporting of our methods will allow replication in future multicenter studies.

### Contribution and Clinical Applicability

The proposed complementary semisupervised home-based exercise training program is of low cost. If shown to have a positive impact on the outcomes of interest, the program will likely impact long-term outcomes such as hospitalizations and 12-month survival and is likely to be implemented in routine-practice cancer exercise or pulmonary rehabilitation programs delivered in settings such as outpatient clinics, rehabilitation centers, and community programs.

### Conclusions

The proposed exercise training program as a complementary treatment for patients with lung or head and neck cancer is likely to promote positive effects on the physical and mental health outcomes assessed, over and above any change in the CG, and prevent the reduction of initial cancer treatment dose prescribed, which will likely impact long-term outcomes such as hospitalizations and 12-month survival. If shown to be effective at improving outcomes, the semisupervised exercise training protocol is likely to be implemented in clinical practice across many cancer and pulmonary rehabilitation programs worldwide.

## Appendix

Multimedia Appendix 1 Exercise diary.

## Abbreviations

1STS: 1-minute sit-to-stand test
6MWT: 6-minute walk test
CG: control group
EORTC QLQ-30: European Organization for Research and Treatment of Cancer Core Quality of Life Questionnaire
HRCPP: Regional Cancer Hospital of Presidente Prudente
HRQoL: health-related quality of life
QLQ-LC13: European Organization for Research and Treatment of Cancer Core Quality of Life Questionnaire–Lung Cancer 13
SPIRIT: Standard Protocol Items: Recommendations for Interventional Trials
TG: training group
TUG: time up and go test

## Data Availability

Data sharing is not applicable to this article as no data sets were generated or analyzed during this study.
