# Supplementary material for: Effects of Semisupervised Exercise Training on Health Outcomes in People With Lung or Head and Neck Cancer: Protocol for a Randomized Controlled Trial
Source: JMIR Res Protoc. 2023 May 24;12:e43547. doi: 10.2196/43547 (PMC10248778; doi:10.2196/43547)
Supplement: Multimedia Appendix 1 [file resprot_v12i1e43547_app1.pdf]

Additional file: Exercise diary.

EXERCISES DIARY - WALK

Patients' name:___________ Week day: ____________ Date:___________

Walk:

- Did you walk today? [ ] Yes [ ] No
- Which were your oxygen saturation before the walk?_____%
- Which were your oxygen saturation after the walk?_____%

- In case you did not walk, why did you not?

[ ] I had a fever. Which was the temperature? _______
[ ] I had an infection.
[ ] I was mentally confused.
[ ] Another reason: ____

Answer the questions below if you have performed your walk today:

**You need to walk for _________ minutes every day and feel [ ] light [ ] moderate shortness of breath.**

- If you have done the walk, how many minutes did you perform it?
  I walked during __________ minutes.
- How many steps your step counter recorded after your walk?
  I walked __________ steps.
- How was your shortness of breath on Borg Scale after the walk?_________
- How was your fatigue on Borg Scale after the walk?_________

ANSWER IN THE DAY FINAL BEFORE SLEEP:
How many steps your step conter is recording now?____________

Congrats!

Keep going, you are doing well!!

EXERCISES DIARY - STRENGHT EXERCISES

Patients' name:___________ Week day: ____________ Date:___________

- Did you perform your exercises today? [ ] Yes [ ] No

- In case you did not walk, why did you not?

[ ] I had a fever. Which was the temperature? _______
[ ] I had an infection.
[ ] I was mentally confused.
[ ] Another reason: ____

- Which were your oxygen saturation before the walk?_____%
- Which were your oxygen saturation after the walk?_____%

Answer the questions below if you have performed the exercises today:

Exercise 1:

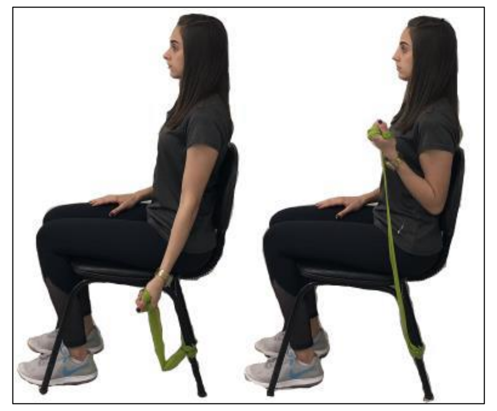

**You need to do this exercise ____ sets of _____ repetitions twice a week.**

- Did you perform this exercise today? [ ] Yes [ ] No
- How many times did you perform this exercise? ______ sets of _____ repetitions.

Exercise 2:

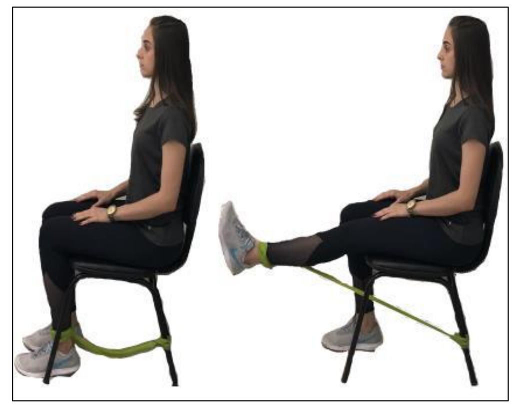

**You need to do this exercise ____ sets of _____ repetitions twice times per week.**

- Did you perform this exercise today? [ ] Yes [ ] No
- How many times did you perform this exercise? ______ sets of _____ repetitions.

Exercise 3:

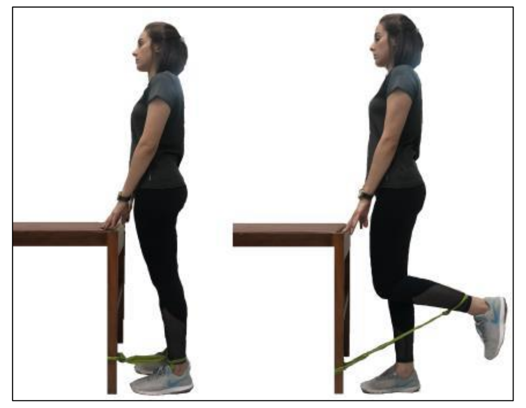

**You need to do this exercise ____ sets of _____ repetitions twice a week.**

- Did you perform this exercise today? [ ] Yes [ ] No
- How many times did you perform this exercise? ______ sets of _____ repetitions.

Congrats!

Keep going, you are doing well!!

Source: Authors own elaboration.
